# Single‐Balloon Endoscopy–Assisted Magnetic Compression Anastomosis for Refractory Biliary Anastomotic Stricture After Hepaticojejunostomy

**DOI:** 10.1111/den.70300

**Published:** 2026-10-08

**Authors:** Kenta Yoshida, Takuto Yamaguchi, Hirotake Sakuraba

**Affiliations:** ^1^ Department of Gastroenterology, Hematology and Immunology, Graduate School of Medicine Hirosaki University Hirosaki Japan

**Keywords:** bile duct stricture, magnetic compression anastomosis (MCA), single‐balloon endoscopy

## 1 Brief Explanation

A 77‐year‐old man developed a refractory biliary anastomotic stricture involving the posterior segmental bile duct with bile leakage after left hepatectomy with Roux‐en‐Y hepaticojejunostomy for perihilar cholangiocarcinoma. Both balloon‐assisted endoscopic and percutaneous approaches failed to achieve guidewire passage across the severely obstructed anastomosis, and internal drainage was not possible despite repeated attempts (Figure 1a–c). Therefore, percutaneous transhepatic biliary drainage (PTBD) was required for biliary decompression. Because the length of the obstructed segment was short (approximately 1 cm) and reoperation was considered highly invasive, magnetic compression anastomosis (MCA) was selected as an alternative treatment [1, 2, 3]. MCA was performed using a combined percutaneous and endoscopic approach assisted by single‐balloon endoscopy. A daughter magnet (samarium‐cobalt, 3 × 4 mm) was delivered to the biliary side of the obstruction through a 14‐Fr sheath placed via the PTBD tract. The smaller daughter magnet was selected to allow passage through the 14‐Fr sheath. Subsequently, a parent magnet (samarium‐cobalt, 5 × 6 mm) was delivered to the enteric side of the hepaticojejunostomy using single‐balloon endoscopy with an overtube. The two magnets were carefully aligned under fluoroscopic guidance until successful coupling was confirmed (Figure 2a,b; Video 1). Nineteen days after MCA, cholangiography showed contrast flow into the intestinal tract, indicating successful fistula formation (Figure 2c). The coupled magnets were retrieved endoscopically, and internal biliary drainage was achieved without procedure‐related adverse events or the need for surgical reintervention (Video 2).

**FIGURE 1:**
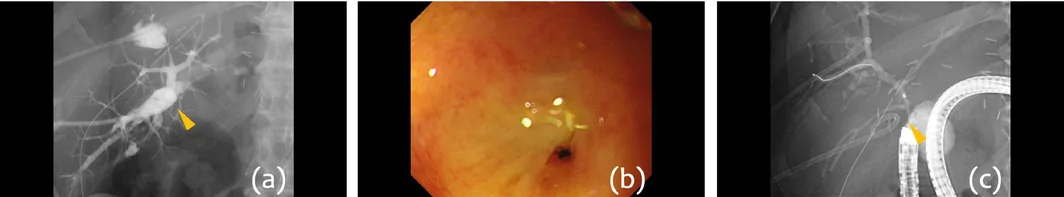
(a) Cholangiography via the right posterior segmental bile duct showed no contrast passage into the jejunum despite sufficient contrast injection. (b) Endoscopic image of the hepaticojejunostomy showed severe anastomotic stricture; visualization of the posterior segmental bile duct was difficult despite adequate contrast injection. (c) Cholangiography using single‐balloon endoscopy failed to visualize the bile duct from the jejunal side because of severe to complete anastomotic stricture.

**FIGURE 2:**
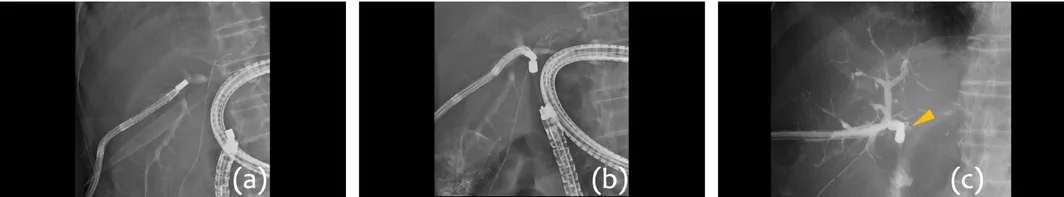
(a) Fluoroscopic image showing the daughter and parent magnets positioned on the biliary and enteric sides of the obstructed anastomosis before coupling. (b) Fluoroscopic image showing successful coupling of the two magnets. (c) Nineteen days after MCA, cholangiography via the percutaneous transhepatic biliary drainage (PTBD) route showed contrast flow into the jejunum, confirming successful fistula formation.

**VIDEO 1:** This video shows the technical tips of single‐balloon endoscopy–assisted magnetic compression anastomosis (MCA) for a biliary anastomotic stricture after hepaticojejunostomy. This technique provides a safe and minimally invasive treatment option for achieving internal biliary drainage when standard endoscopic and percutaneous methods fail. Video content can be viewed at https://onlinelibrary.wiley.com/doi/10.1111/den.70300.

**VIDEO 2:** This video shows safe endoscopic retrieval of the coupled magnets after MCA. Video content can be viewed at https://onlinelibrary.wiley.com/doi/10.1111/den.70300.

MCA is not widely available and typically requires institutional approval and multidisciplinary collaboration, including coordination with experienced specialists. However, this case demonstrates that single‐balloon endoscopy–assisted MCA may be considered as a potential treatment option for carefully selected patients with severely or completely obstructed biliary anastomotic strictures after hepaticojejunostomy when conventional endoscopic and percutaneous techniques fail [4].

## Author Contributions

Dr. Yoshida conceptualized and drafted the manuscript, and performed endoscopic examinations. Dr. Yamaguchi drafted the manuscript. Dr. Sakuraba supervised the study, drafted the manuscript, and made intellectual revisions to the manuscript. All the authors have read and approved the final version of the manuscript.

## Funding

The authors have nothing to report.

## Ethics Statement

The authors have nothing to report.

## Conflicts of Interest

The authors declare no conflicts of interest.

## Data Availability

The data that support the findings of this study are available on request from the corresponding author. The data are not publicly available due to privacy or ethical restrictions.
